# Transcriptome and proteomic analysis of mpox virus F3L-expressing cells

**DOI:** 10.3389/fcimb.2024.1354410

**Published:** 2024-02-13

**Authors:** Yihao Wang, Junzhe Zhang, Mingzhi Li, Mengle Jia, Lingdi Yang, Ting Wang, Yu Wang, Lumei Kang, Meifeng Li, Lingbao Kong

**Affiliations:** ^1^ Institute of Pathogenic Microorganism, Jiangxi Agricultural University, Nanchang, Jiangxi, China; ^2^ Nanchang City Key Laboratory of Animal Virus and Genetic Engineering, Jiangxi Agricultural University, Nanchang, Jiangxi, China; ^3^ College of Bioscience and Engineering, Jiangxi Agricultural University, Nanchang, Jiangxi, China

**Keywords:** transcriptome, proteomic, mpox virus, F3L, innate immune signaling pathway

## Abstract

**Background:**

Monkeypox or mpox virus (mpox) is a double-stranded DNA virus that poses a significant threat to global public health security. The F3 protein, encoded by mpox, is an apoenzyme believed to possess a double-stranded RNA-binding domain (dsRBD). However, limited research has been conducted on its function. In this study, we present data on the transcriptomics and proteomics of F3L-transfected HEK293T cells, aiming to enhance our comprehension of F3L.

**Methods:**

The gene expression profiles of pCAGGS-HA-F3L transfected HEK293T cells were analyzed using RNA-seq. Proteomics was used to identify and study proteins that interact with F3L. Real-time PCR was used to detect mRNA levels of several differentially expressed genes (DEGs) in HEK293T cells (or Vero cells) after the expression of F3 protein.

**Results:**

A total of 14,822 genes were obtained in cells by RNA-Seq and 1,672 DEGs were identified, including 1,156 up-regulated genes and 516 down-regulated genes. A total of 27 cellular proteins interacting with F3 proteins were identified by liquid chromatography-tandem mass spectrometry (LC-MS/MS), and 19 cellular proteins with large differences in abundance ratios were considered to be candidate cellular proteins. Gene Ontology (GO) and Kyoto Encyclopedia of Genes and Genomes (KEGG) enrichment analyses showed that the DEGs were significantly enriched in immune-related pathways, including type I interferon signaling pathway, response to virus, RIG-I-like receptor signaling pathway, NOD-like receptor signaling pathway, etc. Moreover, some selected DEGs were further confirmed by real-time PCR and the results were consistent with the transcriptome data. Proteomics data show that cellular proteins interacting with F3 proteins are mainly related to RNA splicing and protein translation.

**Conclusions:**

Our analysis of transcriptomic and proteomic data showed that (1) F3L up-regulates the transcript levels of key genes in the innate immune signaling pathway, such as *RIGI, MDA5, IRF5, IRF7, IRF9, ISG15, IFNA14*, and elicits a broad spectrum of antiviral immune responses in the host. F3L also increases the expression of the FOS and JNK genes while decreasing the expression of TNFR2, these factors may ultimately induce apoptosis. (2) F3 protein interacts with host proteins involved in RNA splicing and protein translation, such as SNRNP70, POLR2H, HNRNPA1, DDX17, etc. The findings of this study shed light on the function of the F3 protein.

## 1 Introduction

Mpox is a viral zoonosis caused by the mpox virus. From April 2022 to June 2022, there were 528 confirmed cases of mpox infection within 2 months from April to June in 2022, across 43 sites of 16 countries (Thornhill et al., 2022). As of November 30 2023, more than 92,000 human cases of mpox had been laboratory-confirmed (https://www.cdc.gov/poxvirus/mpox/response/2022/index.html). Mpox symptoms are comparable to smallpox symptoms, such as fever (typically between 38.5°C and 40.5°C), headache, muscle aches, swollen lymph nodes, and rash. The main areas of incidence are the palms of the hands, the soles of the feet, the groin and the neck (Adler et al., 2022). The virus is usually spread through direct human-to-human contact (sexual or skin-to-skin), and indirect contact (droplets or bedding) (Thornhill et al., 2022). Mpox was first isolated in 1958 in monkeys imported from Africa to Copenhagen, Denmark and the first human case of mpox infection was reported in the Democratic Republic of the Congo in 1970 (Heymann et al., 1998). The geographic range of human mpox transmission extended beyond Africa for the first time in 2003, when 47 cases were detected in the central and western states of the United States (Xiang and White, 2022). According to the ‘2022 -2023 Mpox Outbreak’ presented by the WHO on December 27 2023, 117 countries, areas, or territories reported mpox cases. Among them, 110 countries, areas, or territories reported mpox cases for the first time (https://www.cdc.gov/poxvirus/mpox/response/2022/world-map.html).

Mpox virus (mpox) belongs to Family *Poxviridae*, subfamily *Chordopoxvirinae*, genus *Orthopoxvirus*, Species *Monkeypox virus*. Mpox contains a double-stranded DNA genome within its core, which is approximately 197 kb in length and contains at least 190 open reading frames (ORFs) (Shchelkunov et al., 2002). As an example, the complete genome of one of the mpox isolates, SI2022_S7 (Gen bank ID: ON838178.1), was 197,652 bp long (Khattak et al., 2022). Mpox may commonly invade the body through the oral and respiratory tracts, subsequently infecting the mucous membranes of organs, then gradually infecting some immune cells (e.g., antigen-presenting cells, macrophages, and B-cells) over time, and finally spreading throughout the body from the lymphatics (Lum et al., 2022). Mpox virions use glycosaminoglycans as host receptors. In the early stages of mpox infection, the mpox-encoded proteins are mainly involved in DNA replication and interact with target proteins, the major intracellular modulations include inhibition of the antiviral system and induction of cell cycle arrest. In the terminal stages of mpox infection, these proteins mainly become an important part of the viral assembly. Mpox finally leaves the infected cell through the actin tail or fusion with the cytoplasmic membrane (Hatmal et al., 2022; Kmiec and Kirchhoff, 2022). These mpox-encoded proteins are classified into three categories: (1) entry proteins (which facilitate mpox entry into host cells through receptor binding and membrane fusion) include *M1R, E8L and H3L*; (2) exit proteins (which facilitate the release of mpox copies from host cells) include *A38R, C23R and C18L*; and (3) immunomodulatory proteins (which are essential proteins for modulation of the host cell and immune modulation) include *J2L, F3L and A41L*(https://viralzone.expasy.org/9976).

The F3L is 459 bp and encodes an approximately 17.5 kD size of the F3 protein, an enzyme with a double-stranded RNA binding domain (dsRBD). The results of research on the mpox-encoded proteins are limited. The study by Arndt et al. found that the mpox F3 protein shares 88% sequence identity with vaccinia virus (VACV) E3 protein that was shown to have full IFN resistance *in vitro* (White and Jacobs, 2012; Arndt et al., 2015). VACV-E3L (RNA-binding protein E3) is one of the key IFN resistance proteins of VACV. E3 protein contains a Z-DNA binding domain (Za domain) at the N-terminus and a highly conserved double-stranded RNA-binding domain (dsRBD) at the C-terminus (Szczerba et al., 2022). E3L has critical roles in evading recognition of double-stranded RNA by host receptor proteins (Kim et al., 2023), inhibiting IFN production (Xiang et al., 2002), and suppressing host cell apoptosis (Garcia et al., 2002).

In our research, in order to acquire transcriptomic information and discover the interactions of mpox-F3L with host cells, HEK293T cells were utilized and transfected with pCAGGS-HA-F3L plasmid (or pCAGGS-HA plasmid), harvested at 24h, and respectively subjected to transcriptomic sequencing or liquid chromatography-tandem mass spectrometry (LC-MS/MS). Gene Ontology (GO) and Kyoto Encyclopedia of Genes and Genomes (KEGG) enrichment analyses were utilized to examine differentially expressed genes (DEGs) or potential cell-interacting proteins identified in HEK293T cells that were overexpressing F3 protein.

We hope our findings will provide new insights to understand the pathogen-host interaction and explore therapeutic measures for mpox infection.

## 2 Materials and methods

### 2.1 Cell culture

Cells were cultured in Dulbecco’s modified eagle medium (DMEM) (Solarbio, China) supplemented with 10% fetal bovine serum (FBS) (Yeasen, China) at 37°C and 5% CO_2_. HEK293T cells and vero cells were purchased from the China center for type culture collection.

### 2.2 Amino acid sequence comparison and molecular phylogenetic analysis

The sequence of mpox-F3L was derived from the NCBI data (GenBank: ON563414.3). The sample in NCBI data was derived from the draft mpox virus genome from a confirmed mpox case in Massachusetts, United States, in May 2022. The clade of mpox is the clade IIb. Structural domain analysis was performed using interPro (Paysan-Lafosse et al., 2023). The sample of molecular phylogenetic analysis is the proteins similar to the mpox virus F3 protein: The evolutionary history was inferred by using the Maximum Likelihood method based on the JTT matrix-based model (Jones et al., 1992). Initial tree(s) for the heuristic search were obtained automatically by applying Neighbor-Join and BioNJ algorithms to a matrix of pairwise distances estimated using a JTT model and then selecting the topology with superior log likelihood value. The analysis involved 33 amino acid sequences. All positions containing gaps and missing data were eliminated. There were a total of 100 positions in the final dataset. Evolutionary analyses were conducted in MEGA7 (Kumar et al., 2016).

### 2.3 Plasmids construction

The gene sequence of mpox-F3L was obtained from the mpox gene data (MPXV_USA_2022_MA001) published by NCBI, and the gene synthesis was performed by Beijing Tsingke Biotech Co., Ltd. Primers were designed based on the mpox-F3L gene sequence. The forward primer (F3L-F) contains the EcoRI cleavage site at the 5’ end, while the reverse primer (F3L-R) contains the XhoI cleavage site (**Table 1**, underlined portion). The mpox-F3L gene sequence was amplified using conventional PCR and cloned into pCAGGS-HA, obtaining the recombinant plasmid pCAGGS-HA-F3L. The pCAGGS-HA plasmid was a gift from National Key Laboratory of Agricultural Microbiology at Huazhong Agricultural University (Wuhan, China).

**Table 1:** Primers used for plasmid construction and real-time PCR.

Primer	Sequence (5’→3′)	Usage	Species
F3L-F	5'-CCGGAATTCATGGAACCAGCCACCAGC-3'	pCAGGS-HA-F3L	
F3L-R	5'-CCGCTCGAGCATTTTGATATACGATATTACAAC-3'		
IFNA14-F	5’-GGTTCAGTGTTACCCCTCATCAA-3’	qRT-PCR	Homo sapiens
IFNA14-R	5’-GGGTTTGAGACAGATTACAGCC-3’		
COX7A1-F	5’-ACCGCTTTCAGAACCGAGT-3’	qRT-PCR	Homo sapiens
COX7A1-R	5’-TGTAGACAGTGCCGCCCA-3’		
DUSP8-F	5’-TCGATAAAGCCAAGCTCTCCA-3’	qRT-PCR	Homo sapiens
DUSP8-R	5’-CCTGTCCTTCACGAACCTGT-3’		
IFI44L-F	5’-CTTTCCTAGAGTCTCTGAAGCCAC-3’	qRT-PCR	Homo sapiens
IFI44L-R	5’-CACTCACACGTGGAAGCTGTT-3’		
HSPA6-F	5’-CAGAGGAACGCCACTATCCC-3’	qRT-PCR	Homo sapiens
HSPA6-R	5’-ACTGAGTTCAAAACGCCCCA-3’		
P2RX7-F	5’-CAGCGGAAAGAGCCTGTCAT-3’	qRT-PCR	Homo sapiens
P2RX7-R	5’-CGAAGAAAGAGTTCCCCTGCAA-3’		
DNAH3-F	5’-TGTTGTTTGGAGGGGGAACT-3’	qRT-PCR	Homo sapiens
DNAH3-R	5’-CTCTCCTATCCAGTTGGTCTGA-3’		
IFI44L-F	5’-CCACCAGCATTACTGAACGG-3’	qRT-PCR	Chlorocebus sabaeus
IFI44L-R	5’-GTAGGGAATGTCATCCACGCA-3’		
COX7A1-F	5’-AGAAGCTCTTCCAGGAGGACAA-3’	qRT-PCR	Chlorocebus sabaeus
COX7A1-R	5’-CTGGTCTTAATTCCTGGGGAAGG-3’		

### 2.4 DNA transfection

According to the manufacturer’s instructions, the pCAGGS-HA and pCAGGS-HA-F3L plasmids were respectively transfected into HEK293T cells or Vero cells using jetPRIME^®^ Transfection Reagent (Polyplus Transfection, France) for 24 hours post transfection, part of the transfected cells lysed in Trizol (Vazyme, China) for RNA extraction and quantitative real-time RT-PCR (qPCR); The other part of these cells lysed in RIPA buffer (Solarbio, China) for further western blot analyses and co-immunoprecipitation (co-IP).

### 2.5 RNA isolation and RNA‐seq

RNA-seq was performed to compare gene expression profiles between pCAGGS-HA-F3L transfected HEK293T cells and pCAGGS-HA (empty vector) transfected HEK293T cells. Total amounts and integrity of RNA were assessed using the RNA Nano 6000 Assay Kit of the Bioanalyzer 2100 system (Agilent Technologies, CA, USA). According to the manufacturer’s instructions (Novo Gene, Beijing, China), mRNA was purified from total RNA by using poly-T oligo-attached magnetic beads. Fragmentation was carried out using divalent cations at elevated temperatures in the First Strand Synthesis Reaction Buffer(5X). The first-strand cDNA was synthesized using random hexamers, followed by a second-strand cDNA synthesis. To select cDNA fragments of preferentially 370~420 bp in length, the library fragments were purified with the AMPure XP system (Beckman Coulter, Beverly, USA). Then, for PCR amplification, the PCR product was purified by AMPure XP beads, and the library was finally obtained. After confirmation of the library quality, the different libraries are pooled according to the effective concentration and the target amount of data off the machine, then sequenced by the Illumina Nova Seq 6000. The end reading of 150bp pairing is generated. The image data measured by the high-throughput sequencer is converted into sequence data (reads) by CASAVA base recognition.

### 2.6 Reverse transcription and quantitative real-time PCR

Total RNA was performed from 5 × 10^5^ cells using Trizol (Vazyme, China). reverse transcription was performed by MonScript™ RTIII All-in-One Mix with dsDNase (Monad, China). RT-qPCR was performed using ChamQ Universal SYBR qPCR Master Mix (Vazyme, China). The sequences of the primers used for qPCR are presented in **Table 1**. The PCR reactions were carried out as follows: one cycle of 95°C for 30s, 40 cycles of 95°C for 10s and 60°C for 30s, and one cycle of 95°C for 15s, 60°C for 1 min and 95°C for 15s. Each RT-qPCR test was performed in triplicate, and the total reaction volume was 20 μl containing 10 μl of Universal SYBR Green mixture, 1 μl of cDNA, and a pair of gene-specific primers. The relative expression value of the selected gene was calculated using the 2^-ΔΔCt^ method and the expression level of the β-actin gene was standardized.

### 2.7 Co-immunoprecipitation and Western blot analyses

The pCAGGS-HA and pCAGGS-HA-F3L plasmids were transfected into HEK293T cells, respectively, and these cells were lysed in RIPA buffer to get whole cell lysate (WCL). The WCL was incubated with anti-HA agarose conjugate (Sigma, Germany) at 4˚C overnight, and collected the WCL of flow through. The co-IP samples were obtained by washing the beads three times with PBS. Part of the co-IP samples and the WCL after anti-HA incubate were subjected to western blot analyses. Western blot analyses were performed by 12% sodium dodecyl sulfate-polyacrylamide gel electrophoresis (SDS-PAGE). The protein samples were electro-transferred to a 0.22 μm PVDF membrane (Millipore, USA). Membranes were blocked with 5% (w/v) skim milk-TBST at room temperature for 1 h and incubated overnight with antibodies against HA-tag, followed by incubation with corresponding horseradish peroxidase (HRP) conjugated antibodies (proteintech, China). The signal was detected using a Chemic DOC imaging system (BioRad, USA).

### 2.8 Liquid chromatography-tandem mass spectrometry

According to the manufacturer’s instructions (Oebiotech, Shanghai, China). RNA-seq was performed on the co-IP samples. The samples were enzymatically digested into peptides, which were desalted and evaporated to dryness. Separation was performed using a Nano-HPLC liquid phase system (EASY-nLC1200). The sample is loaded by an automatic sampler and adsorbed onto a trap column. Subsequently, separation was performed using an analysis column of 75 μ m × A 150mm (RP-C18, Thermo Inc.), and the flow rate was 300nL/min. The enzymatic products were separated by capillary high-performance liquid chromatography. The mass spectrometry analysis was done with a Q-Exactive HF mass spectrometer (Thermo Scientific).

### 2.9 Data processing

Gene Ontology (GO) enrichment analysis of DEGs and interacting proteins was based on the GO database, implemented by KOBAS (http://kobas.cbi.pku.edu.cn/) (Bu et al., 2021). Kyoto Encyclopedia of Genes and Genomes (KEGG)enrichment analysis of DEGs and interacting proteins was based on the KEGG database, implemented by KOBAS (http://kobas.cbi.pku.edu.cn/) (Bu et al., 2021).

All statistical analyses were performed using Prism (GraphPad, v.8.2.1) and SPSS software (version 21.0). The P value < 0.05 or P value < 0.01 were considered significant and highly significant. Some experiments used Student t-tests to assess statistical significance. The bar graph or scattergram showed the most significantly enriched KEGG terms or GO terms.

## 3 Results

### 3.1 Sequence analysis of mpox-F3L

**Figure 1:**
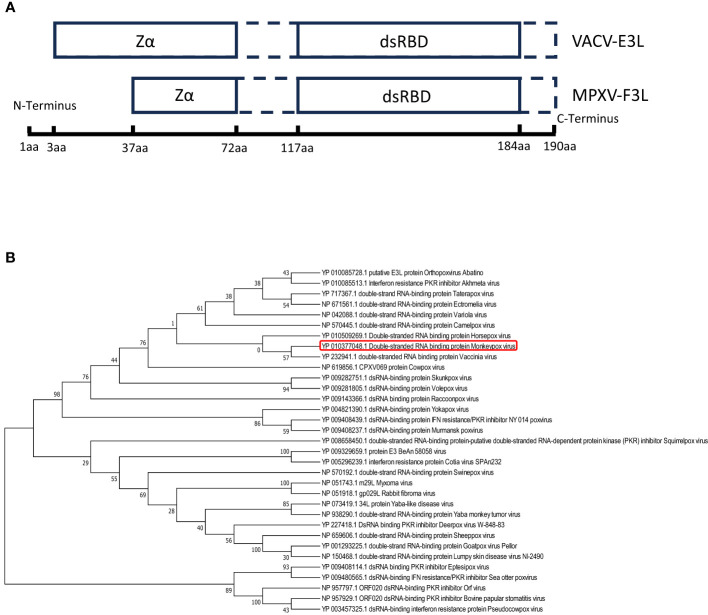
Amio acid sequence analysis of vaccinia virus E3(VACV-E3) and mpox virus F3(mpox-F3) **(A)** Structural domain comparisons of VACV-E3 and mpox-F3. Zα:a Zα-motif-containing domain known to bind left-handed Z-form double-stranded (ds)NA; dsRBD: a C-terminal dsRNA-binding domain known to interact with conventional right-handed A-form double-stranded RNA (dsRNA). **(B)** Molecular phylogenetic analysis of VACV-E3L and mpox-F3L. The tree with the highest log likelihood (-3224.27) is shown. The percentage of trees in which the associated taxa clustered together is shown next to the branches.

The mpox F3 protein is a homologue of the VACV E3 protein (shares 88% identities). Amino acid sequence comparisons between the mpox-F3 and the VACV-E3 show that the F3 protein contains an intact functional dsRNA binding domain (dsRBD) at the C-terminus and an incomplete Z-DNA binding domain (Zα domain) at the N-terminus (Arndt et al., 2015). Compared to E3 protein, F3 protein lacks 37 amino acids in the Zα domain of the N-terminal and has some mutations at the Z-DNA binding domain (**Figure 1A**). To explore whether F3L is conserved among the pox viruses, we performed a phylogenetic analysis for F3L (**Figure 1B**). We searched for the protein (amino acid) sequences similarity to the mpox-F3 protein and constructed a phylogenetic tree and found that most of them were orthopoxvirus proteins. Phylogenetic trees analysis revealed that mpox-F3 and VACV-E3 evolved with shorter divergence and closer kinship in comparison with other proteins. The dsRBD of F3L is conserved in many poxviruses (Vaccinia viruses, Horsepox virus, and Goatpox virus, etc.), which means that our study of mpox-F3L can inform the study of the dsRBD of pox viruses.

### 3.2 Plasmids construction and Western blot analyses of F3 protein

**Figure 2:**
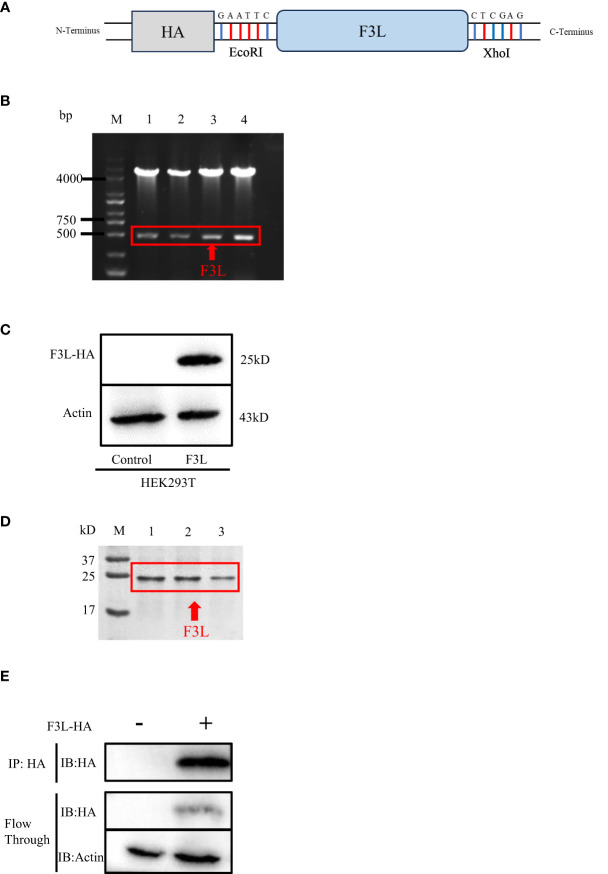
Construction and expression of recombinant F3L in HEK293T cells. **(A)** Model of a cloned fragment. **(B)** Double digestion of the pCAGGS-HA-F3L plasmid. Lane 1-4: F3L recombinants were digested by EcoR I and Xho I. **(C)** Expression of recombinant F3L in HEK293T cells, followed by immunoblot analysis using HA-tag antibodies or anti β-actin antibodies. Lane 1: Transfected with pCAGGS-HA. Lane 2: Transfected with pCAGGS-HA-F3L. **(D)** Identification of purified F3L recombinant protein by SDS-PAGE. M: Trans 180 kD protein molecular standard marker(8-180 kD)1-3: Purification of the recombinant proteins. **(E)** Western blot analysis of the co-IP sample. Flow through: the WCL after anti-HA incubate.

The pCAGGS-HA-F3L plasmid was constructed and verified by DNA agarose gel electrophoresis. The “GAATTC” and “CTCGAG” sequences at both ends of F3L represent EcoRI and XhoI cleavage sites (**Figure 2A**). Double digestion assay was used to verify pCAGGS-HA-F3L plasmid, which showed two prospective bands, one 5000 bp is the pCAGGS vector and one 500 bp is F3L gene in gel (**Figure 2B**). Then, the pCAGGS-HA-F3L plasmid was transfected into HEK293T cells and the F3 protein was detected by HA-tag antibody. The result of the western blot showed that F3 protein (a 25 kD band) can be detected, which suggested that pCAGGS-HA-F3L plasmid was successfully expressed in HEK293T cells (**Figure 2C**). We also used *E. coli* to express and purify F3. The SDS-PAGE results showed a band at around 25 kD, consistent with the western blot results (**Figure 2D**). Partial sample of the co-IP and the WCL after anti-HA incubate (Flow through) were subjected to western blot analyses. The results indicate that most of the F3 protein is adsorbed by anti-HA agarose conjugate (**Figure 2E**).

### 3.3 Identification of differentially expressed genes

**Figure 3:**
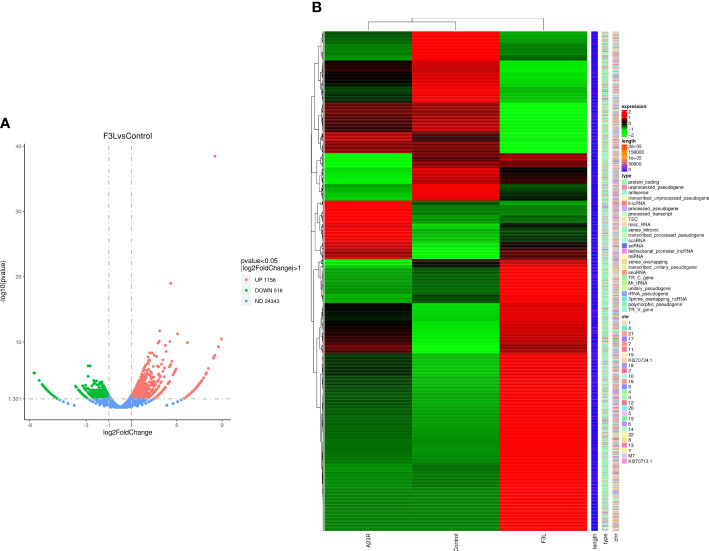
Identification of differentially expressed genes (DEGs). **(A)** The volcano diagram of DEGs. The horizontal axis represents the fold change in gene expression between the experimental and control groups (log2FoldChange). The vertical axis represents the significance of the DEGs between the experimental and control groups (-log10padj or -log10pvalue). Up-regulated genes are shown as red dots. Down-regulated genes are shown as green dots. Threshold lines for DEGs screening criteria are indicated by blue dashed lines. **(B)** Thermal polymerization map of DEGs. The horizontal coordinate represents the sample name. The vertical coordinates on the left represent the cluster analysis. The vertical coordinates on the right represent gene names. The heatmap specifies the length of each gene (length), categorizes its functions (type), and determines its position in the chromosome (chr). The red color in the middle of the heatmap represents high expression, and the green color represents low expression.

RNA-Seq transcriptomics technology is an efficient and rapid way to study genetic transcription and regulation. To determine the effect of F3 protein on the transcriptome of HEK293T cells, RNA-seq was performed to compare gene expression profiles between pCAGGS-HA-F3L transfected cells (experimental group) and pCAGGS-HA transfected cells (control group). The volcano plot provides an overview of the DEGs. Red and green dots in the volcano plot represented the up-regulated and down-regulated DEGs, respectively. There were 1672 differentially expressed genes between the groups of F3L and controls. Compared to the control group, the F3L group had 1156 up-regulated DEGs and 516 down-regulated DEGs (**Figure 3A**). Heatmap data showed the difference in gene expression between the F3L group and the control group (**Figure 3B**). These data suggest that the expression of the F3 protein can affect the expression of multiple genes and elicit significant cellular responses.

### 3.4 Functional enrichment analysis of DEGs

**Figure 4:**
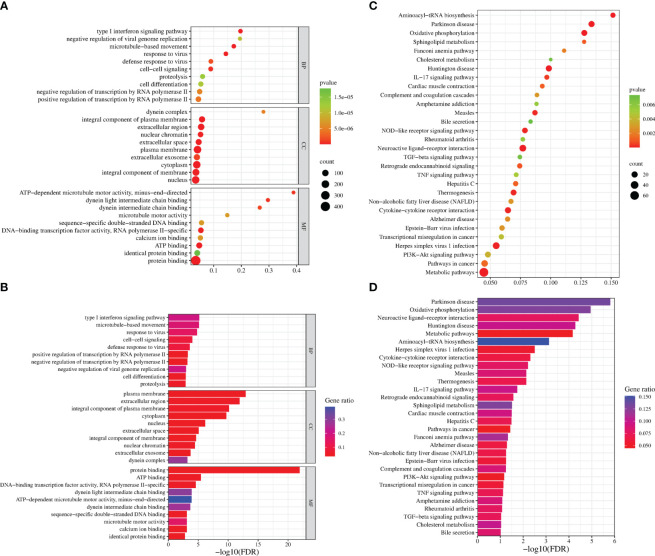
Functional enrichment analysis of DEGs. The scatter plot **(A)** and the histogram **(B)** of GO analysis. The scatter plot **(C)** and the histogram **(D)** of KEGG analysis. The vertical axis represents the top thirty terms with the most significance. The horizontal axis represents the gene ratio **(A, C)** /-log_10_ (FDR) level **(B, D)**. Count: the number of DEGs. Gene ratio: the ratio of DEG number to background gene number. P-value/-log_10_ (FDR): indicators of the significance of the term, the smaller the p-value/the bigger -log_10_ (FDR), the more significant the term.

GO analysis was used to analyze the potential biological functions of DEGs. The results of GO analysis showed that DEGs were categorized into three groups, including biological process (BP), cellular component (CC), and molecular function (MF). Functional analysis revealed that 71 GO terms in the biological process category, among which are the type I interferon signaling pathway (GO:0060337), response to viruses (GO:0009615), regulation of transcription by RNA polymerase II (GO:0006357), and intercellular signaling (GO:0007267). There are35 GO terms in the cellular component category, among which are the cellular component category (GO:0016021 and GO:0005887), cell membrane (GO:0005886, GO:0005887, and GO:0016021) and extracellular regions (GO:0005576, GO:0005615 and GO:0070062). There are33 GO terms in the molecular functions category that were determined to be significantly enriched, among which are the protein-binding (GO:0005515), ATP-binding (GO:0005524), and DNA/RNA-binding related terms (GO:0003677 and GO:0003725) (P < 0.05) (**Figure 4A**). These pathways also had smaller padj values and lower probability of false positives (**Figure 4B**). The pathways with regulated innate immunity and apoptosis screened by GO analysis were also reflected in the KEGG analysis, including RIG-I-like receptor signaling pathway (hsa04622), NOD-like receptor signaling pathway (hsa04621), Toll-like receptor signaling pathway (hsa04620), Cytokine-cytokine receptor interaction (hsa04060), TNF signaling pathway (hsa04668), and NF-kappa B signaling pathway (hsa04064) in KEGG pathways (**Figures 4C, D**). Expectedly, most of the immune-associated GOs and KEGGs appeared to be up-regulated (**Table 2**). These results implied that F3L may affect some immune and apoptosis-related pathways.

**Table 2:** Table of gene enrichment of related pathways.

Terms	Significantly upregulated genes												
**RIG-I-like receptor signaling pathway**	IRF7	IFIH1	ISG15	RIGI	IFNA14								
**NOD-like receptor signaling pathway**	NAIP	CXCL3	IRF7	CCL5	JUN	IRF9	BIRC3	GSDMD	OAS1	CARD9	OAS2	TRPC7	IFNA14
**Toll-like receptor signaling pathway**	CTSK	IRF7	IRF5	CCL5	FOS	JUN	IFNA14						
**Cytokine-cytokine receptor interaction**	EDA2R	CXCL3	CX3CR1	AMH	IL11	CCL5	IL17F	TNFRSF10C	INHBA	NODAL	TNFRSF12A	IFNA14	
**TNF signaling pathway**	CEBPB	CXCL3	FOS	JUN	CCL5	BIRC3	BCL3						
Terms	Significantly downregulated genes												
**RIG-I-like receptor signaling pathway**													
**NOD-like receptor signaling pathway**	P2RX7												
**Toll-like receptor signaling pathway**													
**Cytokine-cytokine receptor interaction**	AMHR2	TNFRSF1B	CXCR4	IL27RA	IL4	BMP5	TNFRSF9						
**TNF signaling pathway**	TNFRSF1B												

### 3.5 Analysis of quantitative real-time PCR

**Figure 5:**
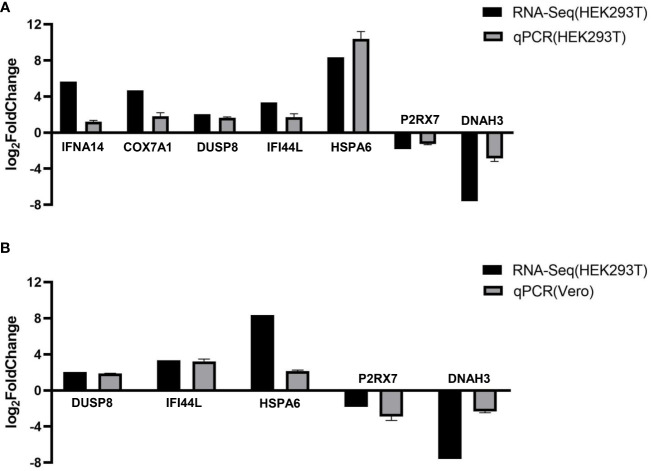
RT-qPCR verified the expression of seven genes. We examined the gene expression levels of *IFNA14, COX7A1, DUSP8, IFI44L, HSPA6, P2RX7*, and *DNAH3* by RT-qPCR in HEK293T cells **(A)** or Vero cells **(B)** after the expression of F3 protein. RNA expression levels in each system were normalized to β-actin. The error bars indicate the SD of repeated RT-qPCR. All experiments were conducted independently, at least three times.

To verify the accuracy of RNA-Seq transcriptomics, RT-qPCR was used to detect some DEGs (*IFNA14, COX7A1, DUSP8, IFI44L, HSPA6, P2RX7*, and *DNAH3*). And we transfected pCAGGS-HA-F3L into HEK293T cells (**Figure 5A**) and vero cells (mpox-susceptible cells) (**Figure 5B**). The results showed that the expressions of *IFNA14, COX7A1, DUSP8, IFI44L*, and *HSPA6* were up-regulated, while the expressions of *P2RX7* and *DNAH3* were down-regulated in F3L-transfected cells. The quantitative PCR was used to validate transcriptome data results. And the qPCR data results also illustrate the similarity of HEK293T and vero cells, indicating that the data from transcriptome sequencing was reliable.

### 3.6 Interaction protein analysis of mpox-F3L by liquid chromatography-tandem mass spectrometry

**Figure 6:**
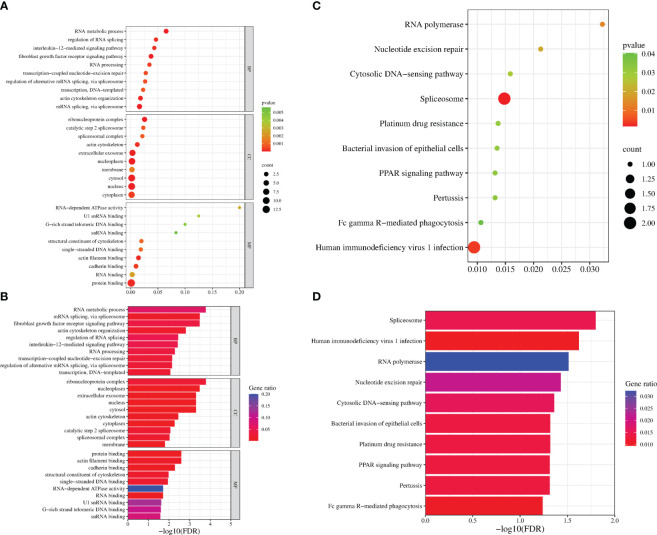
Functional enrichment analysis of the interacting proteins. The scatter plot **(A)** and the histogram **(B)** of GO analysis. The scatter plot **(C)** and the histogram **(D)** of KEGG analysis. The vertical axis represents the top thirty terms with the most significance. The horizontal axis represents the ratio **(A, C)**/-log_10_ (FDR) level **(B, D)**. Count: the number of proteins. Gene ratio: the ratio of protein number to background protein number. P-value/-log_10_ (FDR): indicators of the significance of the term, the smaller the p-value/the bigger -log_10_ (FDR), the more significant the term.

To identify the interacting protein of mpox-F3L, immunoprecipitation(Co-IP) and LC-MS/MS were performed in HEK293T cells. The results were managed by Proteome Discover 2.4 software using the UniProt human database. There were 8 proteins enriched in the control group (cells transfected with pCAGGS-HA) and the experiment group (cells transfected with pCAGGS-HA-F3L). Differently, compared to the control group, the results of the experiment group found 19 specific interacting proteins of mpox-F3L (**Table 3**). KOBAS online software was used to analyze the function of these proteins. The GO analysis enriched biological process, cellular component, and molecular function categories. The biological process category had RNA metabolic process (GO:0003723), mRNA splicing (GO:0000398), regulation of alternative mRNA splicing(GO:0000381), regulation of RNA splicing(GO:0043484), and RNA processing pathways (GO:0006396). The cellular component category had nucleoplasm(GO:0005654) and nucleus pathways (GO:0005634). The molecular function category included protein binding (GO:0005515), DNA binding (GO:0003697), and RNA binding pathways (GO:0003723) (**Figure 6A**). These enriched pathways also had smaller padj values, which means they have lower probabilities of false positives (**Figure 6B**). There were some similar results of GO analysis between RNA-Seq transcriptomics with mass spectrometry analysis, such as RNA-related pathways, nucleus, and DNA/RNA/protein-binding pathways (**Figures 4**, **6**). KEGG analysis was performed to identify pathways of the interaction protein of mpox-F3L, including human immunodeficiency virus 1 infection (hsa05170), bacterial invasion of epithelial cells (hsa05100), and PPAR signaling pathway (hsa03320), these pathways were associated with innate immunity (**Figures 6C, D**).

**Table 3:** The list of interacting protein.

Accession	Gene_Name	Score Sequest HT	Abundance: F3L	Abundance: Control	Ratio of Abundance(F3L/Control)
P0C0S5	H2AZ1	114.72	9906006.25		
Q8WVV4	POF1B	48.81	57664823.88		
P14136	GFAP	35.54	11165431.38		
P60660	MYL6	20.05	2084907.94		
Q99880	H2BC13	9.05	130966.13		
P31943	HNRNPH1	9.03	63684.57		
Q92841	DDX17	8.87	3004703.25		
Q8IYB1	MB21D2	7.99	12719.68		
Q02880	TOP2B	5.39	35568.86		
P52434	POLR2H	4.81	668328.69		
P52907	CAPZA1	4.2	191452.03		
Q16531	DDB1	1.99	61133.12		
P06454	PTMA	1.9	629931.34		
Q9UHD8	SEPTIN9	1.64	16387.94		
P08621	SNRNP70	3.65	1289446.94	55658.35	23.17
P23528	CFL1	10.62	881419.97	53008.09	16.63
Q01469	FABP5	11.88	2773320.63	766334.76	3.62
Q9H853	TUBA4B	61.73	11736878.28	3390780.06	3.46
P09651	HNRNPA1	7.65	499012.50	207664.36	2.40

## 4 Discussion

Monkeypox (mpox) is a zoonotic disease caused by the mpox virus (mpox). Since early May 2022, cases of mpox (monkeypox) have been reported from countries where the disease is not endemic, and continue to be reported in several endemic countries (https://www.who.int/emergencies/situations/monkeypox-oubreak-2022). Mpox poses a major threat to human public health and security. Unfortunately, the underlying infection mechanisms of the mpox virus are not fully clear. To better understand the function of the mpox-F3 protein in the viral lifecycle, we expressed the F3 protein in HEK293T cells, analyzed DEGs in the host cells by RNA-seq and identified substrates of F3 protein using mass spectrometry. Moreover, we analyzed the gene function of F3L and the related cellular signaling pathways involved by GO and KEGG, intending to understand the F3L-host relationship from these data, as well as the possible mechanisms of antiviral immune response in the cells.

Our transcriptomic data revealed the presence of 1,672 DEGs in the host cells, of which 1,156 genes were up-regulated and 516 genes were down-regulated. GO analysis indicated that the DEGs were mainly involved in translation-related pathways. KEGG analysis showed that the DEGs were mainly involved in immune-related pathways such as RIG-I-like receptor signaling pathway, NOD-like receptor signaling pathway, Toll-like receptor signaling pathway, Cytokine-cytokine receptor interaction, TNF signaling pathway, and NF-kappa B signaling pathway, etc. After digging deeper into the DEGs, we found that (1) many key molecules in the innate immune signaling pathway, such as *RIGI, MDA5, IRF5, IRF7, IRF9, ISG15*, and *IFNA14* were shown to be up-regulated in the RNA-seq data. Both *RIGI and MDA5* are pattern recognition receptors that are responsible for virus dsRNA, activate TBK1/IKKϵ or TRIF-dependent pathways, promote phosphorylation of *IRF3 and IRF7* induce IFN-α/β expression (Emralino et al., 2022; Im et al., 2023; Qing and Liu, 2023). *IRF5, IRF7, and IRF9* are positive regulators for interferon expression (Negishi et al., 2018; Jefferies, 2019). (2) Pattern recognition receptors 2’-5’-oligoadenylate synthase 1(OAS1) and 2’-5’-oligoadenylate synthase 2 (OAS2) genes were also found to be up-regulated in the RNA-seq data. The binding of virus dsRNA activates the OAS catalytic center. Then, the activated OAS initiates the activity of RNase L, leading to the degradation of viral RNA (Koul et al., 2020; Schwartz et al., 2020; Yang and Li, 2020). The above data suggest that mpox-F3L can promote the recognition of dsRNA by up-regulating the transcription levels of some key pattern recognition receptor genes, such as *RIGI, MDA5, OAS1, and OAS2*, to trigger their mediated amplification of interferon signaling and elicit the host antiviral immune response.

The transcriptome results provided above appear to differ from the finding that VACV-E3L can inhibit the interferon response (White and Jacobs, 2012). Compared to E3 proteins, F3 proteins differ in the Zα structural domain and the non-structural domain (**Figure 1A**), and thus they differ in function. This may be the reason for their differences in the transcriptome data. It has been reported that VACV-E3L plays an important role in IFN resistance (Jones et al., 1992), blocks PKR recognition of dsRNA, inhibits PKR phosphorylation, and subsequently affects the downstream protein EIF2α, which allows the translation and synthesis of intracellular initiation proteins to proceed normally, thus favoring viral replication (Deng et al., 2008). Although mpox-F3 shared high homology with VACV-E3, amino acid comparisons revealed that the mpox-F3 protein contains only a complete and functional dsRBD at the C-terminal, as does VACV-E3, while missing the first 37 amino acids of its N-terminal Z-DNA binding domain. More critically, the intact N-terminal structural domain of E3 is thought to be required for the suppression of host antiviral immune activation (Arndt et al., 2015). Does the removal of 37 amino acids from the N-terminus of F3 alter its biological function? According to the extant research, we investigated F3 is similar to E3Δ37N (after truncate 37 amino acids of the wild-type E3L at the N-terminus). The E3LΔ37N loses the ability to bind Z-nucleic acid (Z-NA) (Koehler et al., 2017), inhibit phosphorylation of PKR and eIF2 (Langland and Jacobs, 2004; Arndt et al., 2015), and inhibit necroptosis (Koehler et al., 2021) compared to the WT-E3L. These imply that mpox F3L also has these properties. These evidences enlighten us that mpox-F3 cannot target PKR to assume the role of limiting the activation of host antiviral immune response like VACV-E3. The comprehensive transcriptomic data and existing literature reports suggest that our mpox-F3 has a different function from VACV-E3 in the process of antiviral immunization, and that mpox-F3 is capable of eliciting a wide range of antiviral immune response responses in host cells. Further experiments are needed to verify whether this is related to the N-terminal deletion of 37 amino acids.

VACV-E3 is expected to block cell death signaling by binding Z-RNA and Z-DNA through the N-terminal Zα structural domain to inhibit necrotic apoptosis (Koehler et al., 2021) and apoptosis (Kwon and Rich, 2005) in host cells. This prompted us to focus on whether mpox-F3 affects the expression of apoptosis-related factors. We further analyzed the transcriptional data and found that the F3L up-regulates *FOS* and *JNK* genes and downregulates *TNFR2* genes. The TNF (Tumor Necrosis Factor), also known as TNFα, is a cytokine that can directly kill tumor cells and has no significant cytotoxicity to normal cells (Medler and Wajant, 2019), and induces cell survival or apoptosis by binding to two types of receptors (TNFR1 and TNFR2). Rossi AFT et al. have found that the down-regulation of *TNFR2* reduces the expression of host survival genes (NFKB1, CFLIP, and TNFR1), and up-regulates miR-19a and miR-34a to promote apoptosis (Rossi et al., 2022). The TNF signaling activates a variety of downstream molecules, including FOS (Deng et al., 2008) and JNK (Semba et al., 2020), which lead to apoptosis (Yuan et al., 2017). The above data suggest that mpox-F3L can induce apoptosis by up-regulating the FOS and JNK genes and down-regulating the transcriptional level of the TNFR2 gene. It has been reported that neither E3Δ37N (E3 protein with an N-terminal deletion of 37 amino acids) nor E3Δ83N (E3 protein with an N-terminal deletion of 83 amino acids) was able to inhibit apoptosis as wild-type E3 did (Koehler et al., 2021; Szczerba et al., 2022). These evidences also suggests that it may be because of the 37 amino acid deletion at the N-terminus of mpox-F3 compared to VACV-E3 that there is a functional difference between the two proteins in the apoptotic pathway.

The utilization of proteomics in this investigation offers a more intuitive response to the interactions between viral proteins and host cell proteins, somewhat offsetting the limitations of the transcriptome data. With the development of protein research techniques, mass spectrometry identification techniques that are sensitive, efficient, and have high separation capacity and reliability of results have been continuously promoted. Mass spectrometry is currently playing a major role in the study of influenza virus (Downard et al., 2009), Zika virus (Dabrowska et al., 2021), and mpox virus (Lozano et al., 2023). Therefore, in this study we also identified the proteins with potential interactions between mpox-F3 and host cell proteins by mass spectrometry, to find proteins associated with innate immunity and apoptosis. In the mass spectrometry data, we found that mpox-F3 protein bound to SNRNP70, POLR2H, HNRNPA1, and DDX17 proteins (**Table 3**). The SNRNP70 protein not only has a canonical role in mRNA splicing, but is also a key regulator of eukaryotic mRNA length (Deng et al., 2020; Ran et al., 2021). The HNRNPA1 protein is an RNA-binding protein associated with complexes active in a variety of biological processes, such as RNA splicing, trans-activation of gene expression, and protein translation regulation (Siculella et al., 2023). The POLR2H protein is present in all three types of eukaryotic RNA polymerases and is an essential subunit required for nuclear gene transcription (Kang et al., 2006). DDX17 is a member of the DEAD-box deconjugating enzyme family of proteins involved in cellular RNA folding, splicing, and translation (Mao et al., 2021). All of these proteins mentioned above are associated with RNA splicing and protein translation. It has been reported that during apoptosis, the shearing of mRNA precursors and the extra-nuclear transport of RNA are inhibited, and the synthesis of nascent proteins is hindered (Rajani et al., 2012), and undergoes extensive RNA degradation during apoptosis (Liu et al., 2018). Further experiments are now needed to demonstrate whether F3L induces apoptosis by interacting with host proteins to influence processes such as RNA splicing and translation.

As for the viral lifecycle, we found that these genes and proteins can affect the lifecycle of viruses. *RIG-I and MDA 5*, which contain N-terminal caspase recruitment domains, are activated upon the detection of viral RNAs in the cytoplasm of virus-infected cells (Onomoto et al., 2021); *IRF5* deficiency reduces IAV-driven immune pathology and associated inflammatory cytokine production, and finally promotes Influenza Virus-induced inflammatory responses (Forbester et al., 2020); *ISG15* can competitively interact with viral N and P proteins and significantly interfere with their binding to inhibit the peste des petits ruminants virus (PPRV) replication (Tang et al., 2022); *IFN-α14* was identified as the most effective subtype in suppression of hepatitis B virus (HBV) covalently closed circular DNA transcription and HBV e antigen/HBV surface antigen production (Chen et al., 2021). We hypothesize that the up-regulation of these differentially expressed genes (*RIGI, MDA5, IRF5, IRF7, IRF9, ISG15, and IFNA14*) may broadly activate the antiviral immune response of host cells, which may play a role in directly degrading the mpox virus or influencing the replication and assembly of mpox virus. SNRNP70 (U1-70K) plays the primary role in inhibiting polyadenylation of Adeno-associated virus type 5 (AAV5) pre-mRNAs at polyadenylation site [(pA)p], it compromises the translation of the virus (Qiu et al., 2007); HNRNPA1 could inhibit porcine epidemic diarrhea virus (PEDV) replication by degrading viral nucleocapsid (N) protein (Zhai et al., 2023), and also participate in the transcription and replication of a cytoplasmic RNA virus, mouse hepatitis virus (MHV) (Shi et al., 2000); DDX17 interacts with and unwinds Rift Valley fever virus (RVFV) non-coding regions (Nelson et al., 2020). We hypothesized that these RNA splicing and protein translation related-proteins (SNRNP70, POLR2H, HNRNPA1, and DDX17) may play a role in influencing the protein replication and assembly of mpox virus.

Overall, transcriptome sequencing data can conclude that DEGs in host cells are mainly distributed in immune-related pathways, The mass spectrometry data showed that the cellular proteins that interacted with F3 were mainly concentrated in RNA-splicing and protein translation. In summary, F3 proteins play a crucial role in the viral life cycle in two major ways: (1) F3L broadly elicits host antiviral immune response by up-regulating the transcript levels of key genes in the innate immune signaling pathway, such as *RIGI, MDA5, IRF5, IRF7, IRF9, ISG15 and IFNA14*; (2) Interaction of F3 with host proteins related to RNA splicing and protein translation such as SNRNP70, POLR2H, HNRNPA1, and DDX17, as well as inhibition of the transcription of the *TNFR2* gene, up-regulation of the transcriptional levels of the *FOS* and *JNK* genes, and the eventual induction of apoptosis. Taken together, our data provided new insights into understanding the potential responses of immune or apoptosis-related genes against F3L expression.

At present, there are few reports on the pathogenesis and life cycle of mpox virus. The development of vaccines and antiviral drugs against mpox, as well as rapid and precise diagnostic methods, are still the focus of research nowadays. It is essential that we experiment with mpox viruses in the future.

## 5 Conclusions

We investigated reactions of HEK293T cells to transfection with mpox-F3 protein. Transcriptome profiles of protein-transfected cells were examined by RNA-Seq method. In the RIG-I-like receptor signaling pathway, the NOD-like receptor signaling pathway, the Toll-like receptor signaling pathway, and the Cytokine-cytokine receptor interaction as well as immune-related genes showed that F3L broadly elicits host antiviral immune response by up-regulating the transcript levels of key genes, such as *RIGI, MDA5, IRF5, IRF7, IRF9, ISG15 and IFNA14*. The interacting protein profiles of F3 protein-transfected cells were examined by LC-MS/MS method, including SNRNP70, POLR2H, HNRNPA1, and DDX17. These proteins mentioned above are associated with RNA splicing and protein translation. It has been reported that RNA splicing and protein translation are related to cell apoptosis. As well as the transcriptome profiles also showed inhibition of the *TNFR2* gene, up-regulation of the *FOS and JNK* genes, also the eventual induction of apoptosis. The transcriptome-wide RNA-Seq analysis and the interacting protein-wide LC-MS/MS analysis uncovered a large number of novel, interesting candidate pathways and genes for further research. In future work, elucidating the function of newly discovered immune/apoptosis-related genes will be necessary by both molecular and biochemical.

## Data availability statement

The original contributions presented in the study are publicly available. This data can be found here: [NCBI/PRJNA1052933] and [ProteomeXchange/PXD047921].

## Ethics statement

Ethical approval was not required for the studies on humans in accordance with the local legislation and institutional requirements because only commercially available established cell lines were used.

## Author contributions

YHW: Conceptualization, Data curation, Formal analysis, Investigation, Methodology, Project administration, Software, Validation, Writing – original draft, Writing – review & editing. JZ: Data curation, Formal analysis, Writing – original draft. MZL: Methodology, Writing – original draft. MJ: Methodology, Writing – original draft. LY: Methodology, Writing – original draft. TW: Methodology, Writing – original draft. YW: Methodology, Writing – original draft. LMK: Methodology, Writing – original draft. MFL: Formal analysis, Methodology, Project administration, Supervision, Writing – original draft, Writing – review & editing. LBK: Conceptualization, Funding acquisition, Methodology, Project administration, Resources, Supervision, Writing – review & editing.
